# Antibiotic Resistance and Species Profile of *Enterococcus* Species in Dogs with Chronic Otitis Externa

**DOI:** 10.3390/vetsci9110592

**Published:** 2022-10-27

**Authors:** Jun Kwon, Hyoung Joon Ko, Myoung Hwan Yang, Chul Park, Se Chang Park

**Affiliations:** 1 Laboratory of Aquatic Biomedicine, College of Veterinary Medicine and Research Institute for Veterinary Science, Seoul National University, Seoul 08826, Korea; 2 Department of Veterinary Internal Medicine, Jeonbuk National University, Iksan 54596, Korea

**Keywords:** antibiotic resistance, *Enterococcus*, multidrug resistance, otitis externa

## Abstract

**Simple Summary:**

Otitis externa (OE) is a common disease in dogs and can be induced by various causes. After the primary causes that induced the ear canal issue, microbial infections occur secondly. As the main treatment strategies are primary cause correction and antibiotic administration, prolonged treatment is likely to induce the emergence of antibiotic resistance bacteria. Here, we describe the *Enterococcus* bacteria, one of the main infection agents of OE. The bacterial genus showed several species distributions and antibiotic resistance. This fact clarifies the importance of appropriate antibiotic selection and prudent antibiotic administration. As companion animals share lots of space with humans, pathogen transmissions between humans and companion animals are likely to occur. This study contributes not only to treatment strategies for *Enterococcus* infections but can also be used as a comparable index of antibiotic resistance of *Enterococcus* in the future.

**Abstract:**

Otitis externa, a common disease in dogs, has different etiologies. *Enterococcus* is a Gram-positive bacterium that frequently causes opportunistic ear infections. Here, we determined the distribution of *Enterococcus* in canine otitis externa via time-of-flight mass spectrometry and biochemical tests and evaluated their resistance patterns to 10 commonly used antibiotics. Among the 197 *Enterococcus* isolates, *E. faecalis* (48.7%; 96/197) was the most common, followed by *E. faecium* (21.3%; 42/197), *E. casseliflavus* (11.7%; 23/197), *E. hirae* (10.7%; 21/197), *E. avium* (3.6%; 7/197), *E. gallinarum* (2.5%; 5/197), *E. canintestini* (1.0%; 2/197), and *E. durans* (0.5%; 1/197). All isolates were tested for antibiotic resistance using the Kirby–Bauer disc diffusion method. *Enterococcus faecalis* strains were highly resistant to erythromycin (45.8%) and rifampin (34.3%) but were generally susceptible to penicillin class antibiotics. In contrast, *E. faecium* isolates were highly resistant to penicillin class antibiotics (ampicillin, 61.9%; penicillin, 71.4%). Most importantly, *E. faecium* demonstrated high resistance to most of the antibiotics used in this study. Multidrug resistance was found in 28.4% of the isolates (56/197). This study shows prevalence and antibiotics resistance profiles of *Enterococcus* species in canine chronic otitis externa. The results can contribute to establish therapeutic strategies of *Enterococcus* infections and be used as a comparable index of antibiotic resistance of *Enterococcus* in the future.

## 1. Introduction

Otitis externa, inflammation of the external ear canal, has various etiologies and is a relatively common disease with an incidence of 7.5–16.5% in canines [1]. The causes of otitis externa can be broadly divided into primary and secondary causes. The primary causes are those that induce inflammation in the normal ear, such as allergies, autoimmune diseases, and foreign bodies [2,3]. Secondary infections occur when the primary cause alters the composition of the aural environment [2,3].

The external ear canal of dogs with otitis externa is colonized by diverse microorganisms. Among these, Gram-positive enterococci are frequently encountered but are rarely studied as representatives of the external ear canal flora in association with other symbiotic microorganisms. Enterococci are principally commensals of the bowel and are causative agents of opportunistic infections, such as wound infections, mastitis in cattle, and infections of the urethra and ears in dogs [3].

Enterococci are intrinsically resistant to several antimicrobial agents. In humans, multidrug-resistant (MDR) enterococci are among the most important pathogens causing serious nosocomial infections [4]. *Enterococcus faecalis* is reportedly a reservoir of antimicrobial-resistance genes for pathogenic or potentially pathogenic bacteria [5]. Vancomycin is one of the few effective drugs available for the treatment of such infections [6]. However, vancomycin-resistant strains of enterococci have emerged in Europe with the initiation of feeding avoparcin, another glycopeptide, to food-producing animals [6]. In the United States, the injudicious use of vancomycin in human hospitals has resulted in the same effect; that is, an increase in selective pressure resulting in increased colonization of vancomycin-resistant enterococci (VRE) (especially in hospitals) [7]. The prevention and treatment of enterococci infections entails huge expenditures in public health [8,9,10]. Moreover, as farm animals were found to be one of the reservoirs of VRE, enterococci surveillance was expanded to veterinary fields [11,12,13].

Single or repeated exposure(s) to antibiotics could increase the level of resistance in pathogenic bacteria in humans and animals. The level of acquired resistance in bacteria can be considered an indicator of selection pressure due to antibiotic usage in a population and resistance-related problems are expected in pathogens [14]. Regular monitoring of the level of resistance in pathogens and indicator bacteria of the normal flora in both humans and animals is recommended [15]. This monitoring is important because it allows the comparison of the prevalence and evolution of resistance patterns [16]. Knowledge of antibiotic resistance in bacteria in companion animals can help identify potential risks to owners in close contact with companion animals and select optimistic therapeutic drugs in clinical practices. In this study, we aimed to determine the phenotypic resistance patterns of enterococci in the external ear canal of dogs that received antimicrobial treatment.

## 2. Materials and Methods

### 2.1. Enterococcus Isolation and Growth Conditions

*Enterococcus* strains were isolated from external ear canal swab samples collected from dogs with chronic otitis externa from animal hospitals in Seoul and Gyeongsangnam-do, South Korea. The swab samples were maintained in Amies transport medium (YUHAN LAB TECH, Seoul, Korea) until processing. The samples were placed in a 1.5 mL centrifuge tube containing phosphate-buffered saline (PBS) and vortexed vigorously. The supernatants were spread on Columbia blood agar (5% sheep blood; Oxoid, Hamspire, UK) and incubated overnight at 37 °C. Suspected *Enterococcus* colonies were subcultured on fresh tryptic soy agar (TSA; Difco, MI, USA) and incubated overnight at 37 °C. The subculture step on TSA was repeated thrice. After isolating the purified colonies, the isolates were identified via time-of-flight mass spectrometry using a matrix-assisted laser desorption/ionization Biotyper (Bruker Daltonics, Bremen, Germany), according to the manufacturer’s instructions. A total of 197 isolates belonged to the genus *Enterococcus*; however, only 89 strains were identified. The bacteria were stored at −70 °C in tryptic soy broth (Difco) containing 15% glycerol until biochemical tests were performed.

### 2.2. Biochemical Test for Enterococcus Species Confirmation

To confirm and identify the species of all *Enterococcus* isolates, a biochemical test was performed using the API rapid ID 32 STREP kit (bioMérieux SA, Craponne, France) according to the manufacturer’s instructions. The results were visually read by referring to the reading table provided in the product manual. Results were interpreted using an online database (V4.0; https://apiweb.biomerieux.com/ accessed on 20 September 2022). 

### 2.3. Antibiotic Susceptibility Test

The Kirby–Bauer disk diffusion method, as described by the Clinical and Laboratory Standards Institute [17], was used to examine the susceptibility of the *Enterococcus* species to 10 commonly used antibiotics: ampicillin (10 µg), penicillin (10 units), vancomycin (30 µg), doxycycline (30 µg), ciprofloxacin (5 µg), levofloxacin (5 µg), linezolid (30 µg), erythromycin (15 µg), chloramphenicol (30 µg), and rifampin (5 µg). Briefly, overnight cultured colonies of isolates were suspended in sterile PBS to a 0.5 McFarland standard. The suspensions were spread on Mueller–Hinton agar, the antibiotic discs were placed, and incubated at 35 °C for 18 h. For quality confirmation of antibiotic discs, the *Staphylococcus aureus* strain ATCC^®^ 25923 was used. *Enterococcus* isolates resistant to more than three classes of antimicrobials were considered MDR isolates [18].

### 2.4. Statistical Analysis

Differences in resistance prevalence between *E. faecalis* and *E. faecium* were analyzed by the Fischer’s exact test using Microsoft Excel software. A significance level of α 0.05 was used.

## 3. Results

### 3.1. Enterococcus Species Distribution

**Figure 1:**
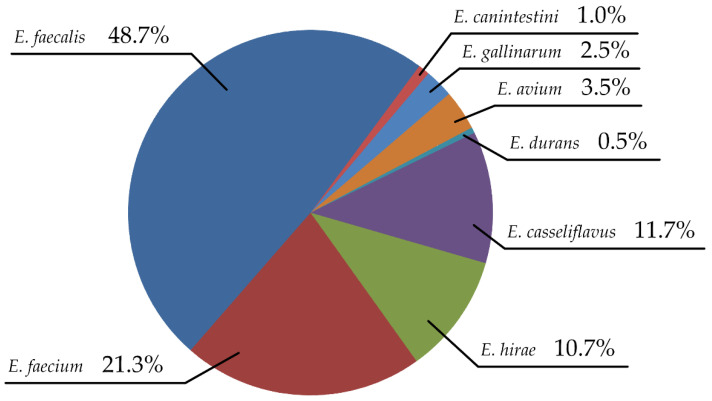
Distribution of *Enterococcus* strains isolated from dogs with chronic otitis externa.

In this study, 214 bacterial colonies were suspected to be *Enterococcus* based on colony morphology. Of these, 197 species were confirmed using time-of-flight mass spectrometry and biochemical tests. Seven species were identified: *E. faecium* (96/197), *E. faecalis* (42/197), *E. casseliflavus* (23/197), *E. hirae* (21/197), *E. avium* (7/197), *E. gallinarum* (5/197), *E. canintestini* (2/197), and *E. durans* (1/197) (Figure 1). *Enterococcus faecalis* was the most frequently isolated species (96 strains; 48.7%), followed by *E. faecium* (42 strains; 21.3%). These two species accounted for approximately 70% of all isolates.

### 3.2. Antibiotic Resistance Profile

Antibiotic susceptibility tests of the *Enterococcus* isolates demonstrated significant resistance of the isolates to different classes of antibiotics. The *E. faecalis* isolates showed the highest rate of resistance against erythromycin (45.8%; 44/96), followed by rifampin (34.4%; 33/96), ciprofloxacin (27.1%; 26/96), levofloxacin (25.0%; 24/96), doxycycline (19.8%; 19/96), linezolid (13.5%; 13/96), chloramphenicol (10.4%; 10/96), and other antibiotics. The large portion of *E. faecium* isolates were resistant to penicillin (71.4%; 30/42), followed by ciprofloxacin (69.0%; 29/42), levofloxacin (66.7%; 28/42), ampicillin (61.9%; 26/42), rifampin (54.8%; 23/42), erythromycin (50.0%; 21/42), doxycycline (38.1%; 16/42), linezolid (23.8%; 10/42), and other antibiotics (Table 1). Five vancomycin-resistant *E. faecalis* (5.2%; 5/96) and *E. faecium* (11.9%; 5/42) strains were identified.

Other *Enterococcus* species showed a relatively low ratio of antibiotic resistance. *E. hirae* isolates were resistant to rifampin in 28.5%; 6/21, followed by linezolid (23.8%; 5/21), doxycycline (19%; 4/21), and ciprofloxacin (19%; 4/21). *E. casseliflavus* strains were mostly resistant to penicillin, ciprofloxacin, linezolid, and rifampin in a 13% ratio (3/23). The other *Enterococcus* species strains also showed resistant strains to several antibiotics.

**Figure 2:**
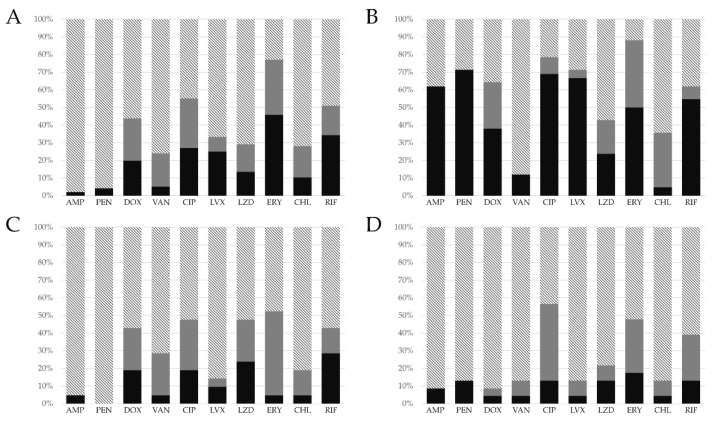
Antibiotic resistance profiles of *Enterococcus* strains isolated from dogs with otitis externa. (**A**) *E. faecalis*. (**B**) *E. faecium*. (**C**) *E. hirae*. (**D**) *E. casseliflavus*. AMP, ampicillin; PEN, penicillin; DOX, doxycycline; VAN, vancomycin; CIP, ciprofloxacin; LVX, levofloxacin; LZD, linezolid; ERY, erythromycin; CHL, chloramphenicol; and RIF, rifampin. Striped, susceptible; gray, intermediate; and black, resistant.

The antibiotic susceptibility test results of four *Enterococcus* species that accounted for over 10% of the total number of isolates were categorized as resistant, intermediate, and susceptible (Figure 2). *Enterococcus faecalis* showed a high percentage of intermediate resistance to erythromycin (31.2%; 30/96), followed by ciprofloxacin (28.1%; 27/96), doxycycline (23.9%; 23/96), and vancomycin (18.7%; 18/96). *Enterococcus faecium* showed a high intermediate resistance rate to erythromycin (38.1%; 16/42). Other *Enterococcus* species showed a relatively low percentage of antibiotic resistance. 

A total of 16 vancomycin-resistant *Enterococcus* isolates (5 *E. faecalis*, 5 *E. faecium*, 2 *E. avium*, 2 *E. gallinarum*, 1 *E. casseliflavus*, and 1 *E. hirae*) were identified, and 27 bacterial strains demonstrated intermediate vancomycin resistance.

**Figure 3:**
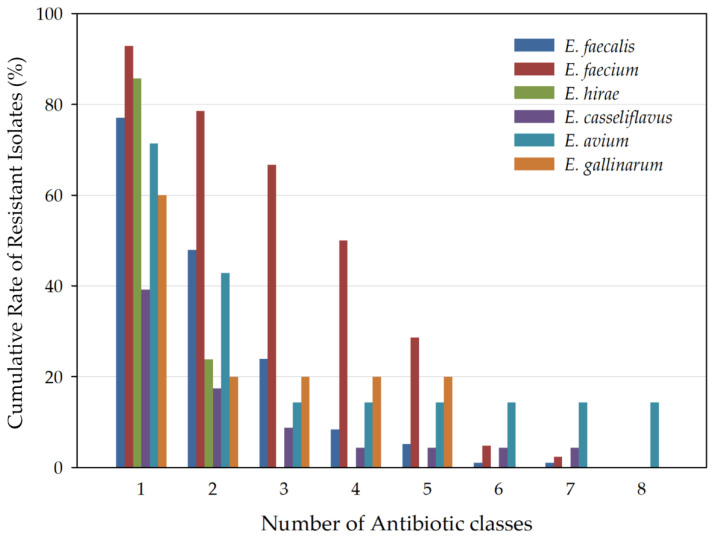
Cumulative percentages of multidrug resistance in *Enterococcus* isolates of six species. *E. faecalis* (23/96; 23.9%), *E. faecium* (28/42; 66.7%), *E. avium* (1/7; 14.3%), *E. casseliflavus* (2/23; 8.7%), *E. canintestini* (1/2; 50%), and *E. hirae* (0/21; 0%).

Multidrug-resistance features of the *Enterococcus* isolates are presented for each species (Figure 3), showing the cumulative percentage of strains that were resistant to one or more antibiotic classes. A total of 28.4% (56/197) of the isolates were found to be MDR. Multidrug resistance was observed more in *E. faecium* than in *E. faecalis*. Among *E. faecalis* isolates, 23/96 (23.9%) were designated as MDR strains. However, 28/42 (66.7%) of *E. faecium* isolates were MDR. *E. faecium* significantly showed a high resistance rate in four antibiotics classes, including penicillin, tetracycline, quinolone, and rifampin (*p* < 0.05). MDR isolates were also presented in the other *Enterococcus* species, *E. avium* (1/7; 14.3%), *E. casseliflavus* (2/23; 8.7%), and *E. canintestini* (1/2; 50%).

## 4. Discussion

*Enterococcus*, along with *Staphylococcus*, is an opportunistic pathogenic genus frequently detected in the external ear canal of dogs [19]. Several studies have reported antibiotic resistance in enterococci; most have focused on the composition and resistance patterns of *Enterococcus* collected from fecal, food, and environmental samples [20,21,22,23]. In some studies on *Enterococcus* from the canine ear canal, *E. faecalis* was the most dominant species [19,24,25]. Similarly, in this study, *E. faecalis* was the predominant species. This study is one of the few to include results on the proportion of other species such as *E. hirae*, *E. casseliflavus*, *E. avium*, *E. gallinarum*, *E. canintestini*, and *E. durans*, in addition to the major *Enterococcus* strains collected from dog ears.

*E. hirae* infections have been reported as exceedingly rare cases in human medicine, but the bacterial species has been regarded as a probable causative agent in veterinary medicine [26,27,28,29,30,31,32]. The infections were reported to induce septicemia, enteritis, and endocarditis in animals, such as chickens, rats, dogs, and pigs [24,25,31,32]. Although *E. hirae* is not a main infectious microbe, infection cases in the immunocompetent have been reported, and these cases were severe and life-threatening. 

*E. gallinarum* and *E. casseliflavus* are intrinsically vancomycin-resistant enterococci (VRE) [33,34,35,36,37]. These species possess the glycopeptide resistance gene C (*vanC*) in a highly conserved genome region [33,34]. These species are resistant to low levels of vancomycin due to the vanC gene [33,34]. In the case of otitis externa, this resistance feature can be crucial. Considering the low-level antibiotic resistance, the target site must be reached at a sufficient antibiotic concentration for effective treatment. However, because high antibiotic concentrations are difficult to maintain in ear canals, from the perspective of treatment strategy, these antibiotic-resistant bacteria can likely contribute to resistance arousal and bacterial reinfection.

The resistance patterns of the strains to 10 commonly used antibiotics belonging to eight classes (penicillins, glycopeptides, macrolides, tetracyclines, fluoroquinolones, phenicols, oxazolidinones, and anamycins) were examined. The prevalence of *E. faecalis* was higher than that of *E. faecium*. However, *E. faecium* had the highest antibiotic resistance among the species. The resistance percentage of *E. faecium* to four antibiotic classes was significantly higher than that of *E. faecalis* (penicillin, tetracycline, quinolone, and rifamycin classes; *p* < 0.05), which is in accordance with Huycke et al. (1998) [38]. Huycke et al. (1998) mentioned an alarming increase in the antibiotic resistance level of *E. faecium*. Recent research has also represented the distributions of *Enterococcus* species and antibiotic resistance profiles [19,39,40]. Each study showed different results in *E. faecalis* (21.3–87.7%) and *E. faecium* (12.1–59.6%) prevalence and resistance patterns of isolates, but consistency in the significant resistance rate of *E. faecium* (over 40%) against the penicillin class.

Previous research has found that *E. faecium* has a high rate of antibiotic resistance [41]. In particular, the studies focused on its resistance to cell wall inhibitory agents. According to reports, approximately 30% of clinical isolates of *E. faecium* were resistant to penicillin and even combinations with aminoglycoside antibiotics [41,42]. Compared to this study, the resistance ratio towards penicillin class antibiotics is twice as high as reported. That is because not only of the characteristics of *E. faecium* species, but also of repetitive and prolonged antibiotic treatment.

Vancomycin resistant enterococci (VRE) are important microorganisms in clinical practice [6,43,44]. Therapeutic alternatives against VRE are limited to recently introduced antibiotics, such as daptomycin, linezolid, and quinupristin/dalfopristin [43]. Considering the intermediate antibiotic resistance, the target site must be reached at the maximum antibiotic concentration for effective treatment [45,46]. However, high drug concentrations are difficult to maintain in ear canals because ear canal skin has been damaged, and exudates and waxy materials in the ear canal hinder achieving enough concentrations. From the perspective of treatment strategy, intermediate antibiotic-resistant bacteria can likely contribute to bacterial reinfection [45,46].

*Enterococcus faecalis* and *E. faecium* showed apparent differences in penicillin class antibiotic (ampicillin and penicillin) resistance (*p* < 0.05). *Enterococcus faecalis* showed a resistance percentage of 2.1% to ampicillin and 4.1% to penicillin, whereas *E. faecium* showed a resistance percentage of 61.9% to ampicillin and 71.4% to penicillin. In a previous study in humans, infections by ampicillin-resistant *Enterococcus* have increased [47], and this is alarming as *E. faecium* causes bacteremia with a higher mortality rate than *E. faecalis* [47,48]. Ampicillin-resistant enterococci were found to be carried not only by humans but also by companion animals [12,49,50,51]. Several studies described a high prevalence of ampicillin-resistant enterococci in companion animals, and even lineages between human infections [49,50].

Of the 197 isolates, 56 (28.4%) were found to be MDR strains. Previous studies mainly discussed MDR *Staphylococcus* and *Pseudomonas* species as the main pathogens of otitis externa [25,52,53]. However, some studies, including the present study, demonstrated *Enterococcus* bacteria and their multidrug resistance patterns [19,53]. These findings provide a warning and highlight the emergence of antibiotic resistance in another bacterial genus. The MDR *Entericoccus* can cause infections, sometimes outbreaks, and prolong therapeutic lapses depending on infection control agents. These serial steps cause high mortality and high costs in medical care. Nelson et al. (2022) showed MDR infections cost nearly USD 1.9 billion and resulted in over 10,000 deaths in the United States in 2017 [54]. Therefore, it is important to monitor the resistance of *Enterococcus* species as an indicator of antibiotic abuse or misuse.

Companion animals have a social function in modern times. They share living environments with human beings, and participate in several activities. However, as human and companion animal relations are getting closer, it is more likely to transmit zoonotic diseases [55,56]. In terms of One Health, the zoonotic aspect is becoming a big issue. The main topic in this section was vector-borne infectious diseases, but recently pathogenic bacterial transmission has received attention [57]. Antimicrobial resistance bacterial infections in companion animals are examples of this [58]. Therefore, it is important to assess microbiological risks. The risks can be transmission of infectious agents and resistance gene transfer [59].

In this study, we evaluated the antimicrobial resistance patterns of *Enterococcus* species isolated from dogs with chronic otitis externa. With the emergence of antibiotic resistance being one of the greatest risks to public health, it is important to clarify the antibiotic resistance of microbes and establish appropriate therapeutic strategies. The results of this study can not only contribute to treatment strategies for *Enterococcus* infections but also be used as a comparable index of antibiotic resistance for *Enterococcus* in the future.

## Figures and Tables

**Table 1:** The prevalence of antimicrobial resistance in *Enterococcus* strains isolated from chronic otitis externa in dogs.

Antibiotics	*E. faecalis*	*E. faecium*	*E. casseliflavus*	*E. hirae*	*E. avium*	*E. gallinarum*	*E. canintestini*	*E. durans*
	n = 96	n = 42	n = 23	n = 21	n = 7	n = 5	n = 2	n = 1
AMP	2 (2.1%)	26 (61.9%)	2 (8.7%)	1 (4.8%)	2 (28.5%)	1 (20%)	1 (50%)	-
PEN	4 (4.2%)	30 (71.4%)	3 (13%)	-	2 (28.5%)	1 (20%)	-	-
DOX	19 (19.8%)	16 (38.1%)	1 (4.3%)	4 (19%)	1 (14.3%)	-	-	-
VAN	5 (5.2%)	5 (11.9%)	1 (4.3%)	1 (4.8%)	2 (28.5%)	2 (40%)	-	-
CIP	26 (27.1%)	29 (69%)	3 (13%)	4 (19%)	1 (14.3%)	1 (20%)	1 (50%)	-
LVX	24 (25%)	28 (66.7%)	1 (4.3%)	2 (9.5%)	1 (14.3%)	1 (20%)	1 (50%)	-
LZD	13 (13.5%)	10 (23.8%)	3 (13%)	5 (23.8%)	2 (28.5%)	-	-	-
ERY	44 (45.8%)	21 (50%)	4 (17.4%)	1 (4.8%)	3 (42.8%)	1 (20%)	-	-
CHL	10 (10.4%)	2 (4.7%)	1 (4.3%)	1 (4.8%)	1 (14.3%)	-	1 (50%)	-
RIF	33 (34.4%)	23 (54.7%)	3 (13%)	6 (28.6%)	2 (28.5%)	2 (40%)	1 (50%)	-

AMP, ampicillin; PEN, penicillin; DOX, doxycycline; VAN, vancomycin; CIP, ciprofloxacin; LVX, levofloxacin; LZD, linezolid; ERY, erythromycin; CHL, chloramphenicol; RIF, rifampin.

## Data Availability

Not applicable.
